# HDMTX-based induction therapy followed by consolidation with conventional systemic chemotherapy and intraventricular therapy (modified Bonn protocol) in primary CNS lymphoma: a monocentric retrospective analysis

**DOI:** 10.1186/s42466-019-0024-2

**Published:** 2019-06-20

**Authors:** Sabine Seidel, Agnieszka Korfel, Thomas Kowalski, Michelle Margold, Fatme Ismail, Roland Schroers, Alexander Baraniskin, Hendrik Pels, Peter Martus, Uwe Schlegel

**Affiliations:** 1 Department of Neurology, Knappschaftskrankenhaus University of Bochum, In der Schornau 23 – 25, D-44892 Bochum, Germany; 2 Department of Hematology and Oncology, Charité Berlin, University of Berlin, Augustenburger Platz 1, D-13353 Berlin, Germany; 3 Department of Hematology and Oncology, Knappschaftskrankenhaus University of Bochum, In der Schornau 23 – 25, D-44892 Bochum, Germany; 4 Department of Neurology, Hospital Barmherzige Brüder, Prüfeninger Straße 86, 93049 Regensburg, Germany; 5 Department of Biostatistics and Clinical Epidemiology, University of Tübingen, Silcherstr. 5, D-72076 Tübingen, Germany

**Keywords:** PCNSL, Methotrexate, Consolidation chemotherapy, Intraventricular therapy, Bonn protocol

## Abstract

**Background:**

To evaluate outcome and toxicity of High-dose methotrexate (HDMTX)-based induction therapy followed by consolidation with conventional systemic chemotherapy and facultative intraventricular therapy (modified Bonn protocol) in patients with primary CNS lymphoma (PCNSL).

**Methods:**

Between 01/2005 and 12/2013 113 patients with newly diagnosed PCNSL presented at our center; 98 of those qualified for HDMTX based chemotherapy, received a modified Bonn protocol and were included in the analysis. The treatment regimen was based on the “Bonn protocol”, but modified by omission of systemic drugs not able to cross the intact blood brain barrier. Intraventricular therapy was postponed until completion of three induction chemotherapy cycles or was replaced by intrathecal liposomal AraC and rituximab was added to induction from 2010 onwards.

**Results:**

Median patient age was 67 years (range 38–83). Complete response/complete response unconfirmed (CR/CRu) was achieved in 59/98 patients (60%), partial response (PR) in 9/98 patients (9%). Twenty-four patients (23%) had progressive disease (PD), 6 (6%) died on therapy. Median progression-free survival (PFS) for all patients was 11.4 months, median overall survival (OS) 29.1 months. A trend to better outcome for intraventricular therapy versus intrathecal liposomal AraC was found in patients < 65 years (HR 0.53 [0.19–1.47] for OS and 0.46 [0.21–1.02] for PFS. Ommaya reservoir infection occurred in 3/33 patients (9%).

**Conclusions:**

The data of this single center experience suggest that the outcome with a modified Bonn protocol was comparable to that of the previous regimen, showed fewer Ommaya reservoir infections and may have a trend for better outcome with intraventricular therapy.

**Electronic supplementary material:**

The online version of this article (10.1186/s42466-019-0024-2) contains supplementary material, which is available to authorized users.

## Background

Primary CNS lymphoma (PCNSL) accounts for about 3% of primary brain cancers [1]. It is a highly aggressive tumor and optimal treatment is not yet defined [2]. PCNSL is highly responsive to radiation, however, whole-brain radiotherapy (WBRT) cannot control the disease if given alone [3]. High-dose methotrexate (HDMTX) is the most active drug in PCNSL. Thus, in the 1990ies, the combination of HDMTX based chemotherapy with WBRT given for consolidation became treatment of choice in PCNSL, but was associated with a high rate of delayed neurotoxicity, particularly in the elderly [4]. No overall survival prolongation was found by adding 45 Gy-WBRT to HDMTX-based primary chemotherapy in a randomized phase III-study [5]. To avoid late neurocognitive decline radiation-free treatment protocols were developed, combining HDMTX with other drugs given systemically and intrathecally. The “Bonn protocol” first published in 2003 [6] resulted in durable responses in a substantial fraction of patients, particularly in the younger, when evaluated in a single arm phase II-trial [6–8]. Systemic HDMTX- and High-dose cytarabine (HDAraC)-based chemotherapy was applied sequentially and combined in each of six treatment cycles with intraventricular therapy (MTX, prednisolone, AraC) via an Ommaya reservoir. Median overall survival (OS) was 50 months and failure free survival (FFS) 21 month [6] without neurotoxicity at long-term follow-up [8]. This protocol has not found broad acceptance due to a 19% rate of Ommaya reservoir infections. On the other hand, a phase II-trial of our group was prematurely stopped, when young patients treated with systemic therapy alone according to the Bonn protocol suffered from early and leptomeningeal relapses [9].

Clinical research in PCNSL is currently focused on defining the optimal consolidation treatment after HDMTX-based induction. With studies exploiting conventional non-cross resistant systemic chemotherapy [10], high dose chemotherapy with autologous stem cell transplantation (HD-ASCT) [11] or dose-reduced WBRT [12], 2-years PFS of 37–77% and 2-years OS of 42–90% have been reported. Two randomized studies compared standard-dose WBRT and HD-ASCT for consolidation in younger patients. No significant difference in 2-years PFS between WBRT and ASCT (80% vs 69%, *p* = 0,17) was found in the IELSG-32 trial [13], but a 24% better 2-years PFS: 86.8% vs. 63.2% was found with HD-ASCT in the French PRECIS trial [14] which, however, was not designed for a direct comparison. In conclusion, an optimal consolidation after HDMTX-based induction therapy has not been established thus far.

Chemotherapy in this series is based on the Bonn protocol [6, 8] and the goal was to preserve its efficacy while reducing its acute toxicity: Cyclophosphamide was replaced by ifosfamide for its better CNS penetration [15]; vincristine und vindesine which are not able to cross the intact blood-brain barrier (BBB) were omitted, and the duration of HDMTX infusion was reduced from 24 to 4 h according to pharmacokinetic considerations [16]. Rituximab was given to all patients treated from September 2010 onwards based on retrospective analyses on its role in PCNSL [17, 18]. However, due to its limited CNS penetration [19] the application of rituximab was restricted to the induction phase where an open BBB is assumed [20]. Most notably, the frequent (4x/week) intraventricular injections were started after three full cycles of induction with initiation of the consolidation phase or were replaced by intrathecal liposomal cytarabine given every 2 weeks. We thus avoided intraventricular therapy during induction, when patients` general condition frequently is compromised due to the higher tumor burden, reduced mobility and concomitant (immunosuppressant) steroids. However, after a Scandinavian consortium had failed to achieve good results with a regimen based on the Bonn protocol for elderly patients, when intraventricular therapy was replaced by intrathecal liposomal AraC [21], we did not apply liposomal AraC in the following. While high dose intravenous MTX application leads to cytotoxic drug concentrations in the cerebrospinal fluid for up to 48 h [22], more sustained drug concentrations for up to 1 week can be achieved with repeated daily intraventricular injections [23].

Thus, depending on patient age and treatment phase, patients were treated differently in this series and a heterogeneously treated group is analyzed. Here we present treatment results obtained with a modified Bonn protocol in 98 patients with a median follow up of more than 7 years. With this report we want to document results achievable in everyday practice with a modified chemotherapy alone regimen in PCNSL.

## Patients and methods

All HIV-negative patients, 18 years and older, presenting at this tertiary care center with newly diagnosed and histologically confirmed PCNSL are offered a modified Bonn protocol if they are able to tolerate HDMTX defined by lack of severe organ dysfunction, in particular renal insufficiency with a glomerular filtration rate below 50 ml/min. Between January 2005 and December 2013 113 patients with newly diagnosed PCNSL presented at our center. In December 2013 an arbitrary cut-off was made to report a minimum follow up of 4 years. Of 113 patients, 7 patients (6%) were unable to receive HDMTX-based chemotherapy due to the following reasons: renal insufficiency (*n* = 4), high age and significant comorbidities (*n* = 2), toxic hepatitis with liver dysfunction, infection (peritonitis) and patient’s refusal in one patient each. Two of these patients were treated with radiotherapy and five patients received best supportive care alone. Four patients received individualized MTX-based protocols for the following reasons: One patient had a ventriculoperitoneal shunt because of subarachnoid hemorrhage years before diagnosis of PCNSL and could not receive intraventricular chemotherapy via an Ommaya reservoir. One patient was admitted in a critically ill status and received only 1 cycle HDMTX monotherapy. Four patients received the original Bonn protocol [6]. Therefore, 98 patients received chemotherapy with a modified Bonn protocol and were included in this analysis. As this was a retrospective analysis on data obtained from the archives of the clinic no written informed consent was obtained. The ethics committee of faculty of medicine of the University of Bochum approved the study.

**Fig. 1:**
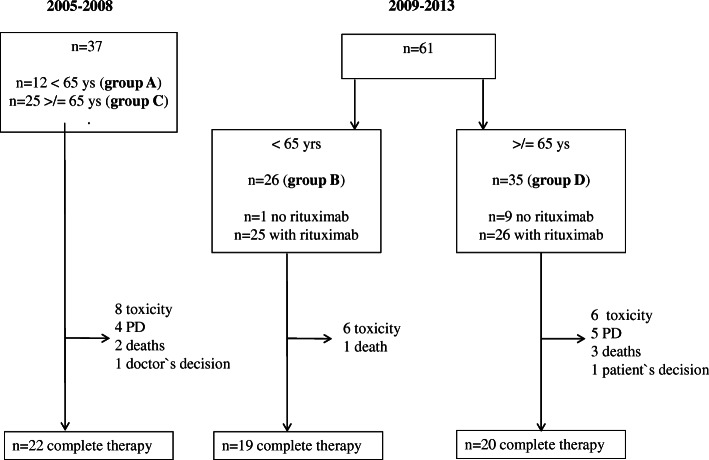
Course of therapy for *n* = 98 patients (group A (< 65 ys 2005–2008) and group C (≥65 ys 2005–2008) received intrathecal liposomal Ara C; group B (< 65 ys 2009–2013) received intraventricular therapy via Ommaya reservoir [25/26 patients]; group D (≥ 65 ys 2009–2013) received no intrathecal therapy [27/35 patients])

Pre-therapy evaluation was performed according to International PCNSL Collaborative Group (IPCG) recommendations [24]. Systemic HDMTX was administered as a 4 h infusion under vigorous hydration and urine alkalization at a dosage of 3–5 g/m^**2**^ body-surface area. From 2005 to 2009 patients < 65 years were given 5 g/m^**2**^ and patients ≥65 years received 3 g/m^**2**^ as in the original Bonn protocol. From 2010 to 2013 patients in general received 4 g/m^**2**^, which was adjusted individually according to glomerular filtration rate. After completion of HDMTX infusion, an intensified leucovorin rescue was performed in case of delayed MTX-clearance. Ifosfamide was given at a dose of 800 mg/m^2^ body-surface area as a 1 h infusion with sodium 2-mercaptoethane sulfonate protection. AraC was given in a dose of 3000 mg/m^2^ body-surface area as a 3 h infusion. Rituximab was given at a dosage of 500 mg/m^2^ body-surface area, a dosage higher than in systemic lymphoma for its poor blood brain barrier penetrance [19]. Intraventricular or intrathecal therapy was applied depending on time of diagnosis as presented in Table 1. From 2005 to 2009 all patients received intrathecal therapy. From 2010 to 2013 only patients < 65 years and 8 “biologically young” patients ≥65 years received intraventricular therapy. Treatment course in all 98 patients is presented in Fig. 1. Response was assessed by contrast-enhanced cMRI scans after 2 to 3 courses and 4 to 6 weeks after completion of treatment and categorized according to the IPCG-criteria (CR = complete response, PR = partial response, PD = progressive disease) with the additional response category “complete response unconfirmed (CRu)”, which allows small enhancing abnormalities in the original tumor localization related to biopsy or focal hemorrhage [24].

**Table 1:** Overview of the intended chemotherapy protocol (* patients treated 2009–2013, ** patients treated 2005–2008, i.v. = intravenous, MTX = methotrexate, AraC = cytarabine, i.th. = intrathecal)

	Day 0	Day 1	Day 2	Day 3	Day 4	Day 5	Day 6
Cycle 1–3 (1 cycle = 2 weeks)							
- Rituximab 500 mg/m^2^ i.v.*	x						
- MTX 3000–5000 mg/m^2^ i.v.		x					
- Ifosfamide 800 mg/m^2^ i.v.			x	x	x		
- Liposomal AraC 50 mg i.th.**			x				
Cycle 4 + 6 (1 cycle = 3 weeks)							
- Cytarabine 3000 mg/m^2^ i.v		x	x				
- Liposomal AraC 50 mg i.th.**				x			
- MTX 2,5 mg + prednisolone 3 mg intraventricular (patients < 65 ys)*				x	x	x	
- AraC 10 mg intraventricular (patients < 65 ys)*							x
Cycle 5 (1 cycle = 2 weeks)							
- MTX 3000–5000 mg/m^2^ i.v.		x					
- Ifosfamide i.v. 800 mg/m^2^ i.v.			x	x	x		
- Liposomal AraC 50 mg i.th.**			x				
- MTX 2,5 mg + prednisolone 3 mg intraventricular (patients < 65 ys)*			x	x	x		
- AraC10 mg intraventricular (patients < 65 ys)*						x	
Cycle 5 was repeated for patients < 65 ys

Data concerning toxicity was classified according to the WHO system [25]. All patients were included in a follow up-program. Within the first 2 years clinical controls, MRI controls and ophthalmological controls were carried out every 3 months, afterwards every 6 months for 3 years and in the following annually. Additional examinations were performed on clinical suspicion only.

Overall survival (OS) was calculated from the date of histologic diagnosis to death of any cause or last date of follow up. Progression free survival (PFS) was defined as the time from date of histologic diagnosis to progression, death of any cause (if progression was not determined) or last date of follow up. OS and PFS were estimated by the Kaplan-Meier method. Log-rank tests were used to compare OS and PFS between groups. Additionally, simple and multiple Cox proportional hazard regression models were calculated and hazard ratios, two-sided 95% confidence intervals, and *p*-values were presented. For each of the non-significant variables results refer to the model in which significant variables and a single non-significant variable was included. The level of significance was 0,05 (two-sided). Analyses were conducted using SPSS version 23.0, SPSS Inc., Chicago, IL.

## Results

### Patients` characteristics and treatment

Pretherapeutic characteristics are summarized in Table 2. Three patients (3%) were considered immunocompromised due to long-term oral steroid therapy for an autoimmune disease (polyarthritis *n* = 2, alveolitis *n* = 1). Thirty-seven patients (12 patients < 65 years [group A] and 25 patients ≥65 [group C]) treated between 2005 and 2008, received 50 mg intrathecal liposomal AraC on day 2 or 3 of each chemotherapy course instead of intraventricular therapy. Of 61 patients treated thereafter, 26 patients were <  65 years (group B). 25/26 patients in group B received intraventricular chemotherapy via an Ommaya reservoir during consolidation (starting with cycle 4 of all cycles). 35 patients treated from 2009 to 2013 were ≥ 65 years (group D), 8 of these patients were treated with intraventricular therapy starting with cycle 4 (since they were considered “biologically younger”) and 27 patients received no intrathecal therapy at all.

**Table 2:** Pretherapeutic patients` characteristics

Age		
- Median (range 38–83)	67	
- < 65	38	39%
- ≥ 65	60	61%
Sex		
- Male	45	46%
- Female	53	54%
Surgery		
- Stereotactic biopsy	60	61%
- Open biopsy	19	19%
- Partial resection	5	5%
- Complete resection	11	11%
- Vitreal cytology	2	2%
- CSF cytology	1	1%
Neuropathologial Diagnosis		
- Diffuse large-cell B-cell lymphoma	92	94%
- Lymphoma, not specified	6	6%
- Other	0	
CSF diagnostic		
- elevated cell count	26/82	32%
- elevated CSF protein	45/80	56%
- lymphoma cells in CSF cytology	10/74	14%
- lymphoma cells in flow cytometry	3/53	6%
Karnofsky-Performance Score		
- median (range 20–100)	60	
- ≥ 70	45	46%
- < 70	53	54%
MSKCC Score		
- ≤ 50 years	9	9%
- > 50 years/KPS ≥ 70	39	40%
- > 50 years/KPS ≥ 70	50	51%
IELSG Score		
- score 0–1	11/74	15%
- score 2–3	40/74	54%
- score 4–5	23/74	31%

All patients treated after September 2010 (25/26 = 96% in group B and 26/35 = 74% in group D) received rituximab. Sixty-one patients (62%) received the complete protocol as planned. Reasons for premature termination were: toxicity in 20 patients (20%), lymphoma progression in 9 (9%), toxicity-related death in 6 (6%), treating physician’s decision (patient’s incompliance) and patient’s decision in 1 patient each (1%).

### Treatment outcome

After completion of therapy CR/CRu was achieved in 59 patients (60%) and PR in 9 patients (9%) accounting for an overall response rate of 69%. Twenty-four patients (23%) had progressive disease (PD) and 6 (6%) died on therapy. Median follow up was 84,4 months (range 4.8–135.9 months). Two patients were lost to follow up.

Median PFS for all patients was 11.4 months, median OS was 29.1 months.

**Fig. 2:**
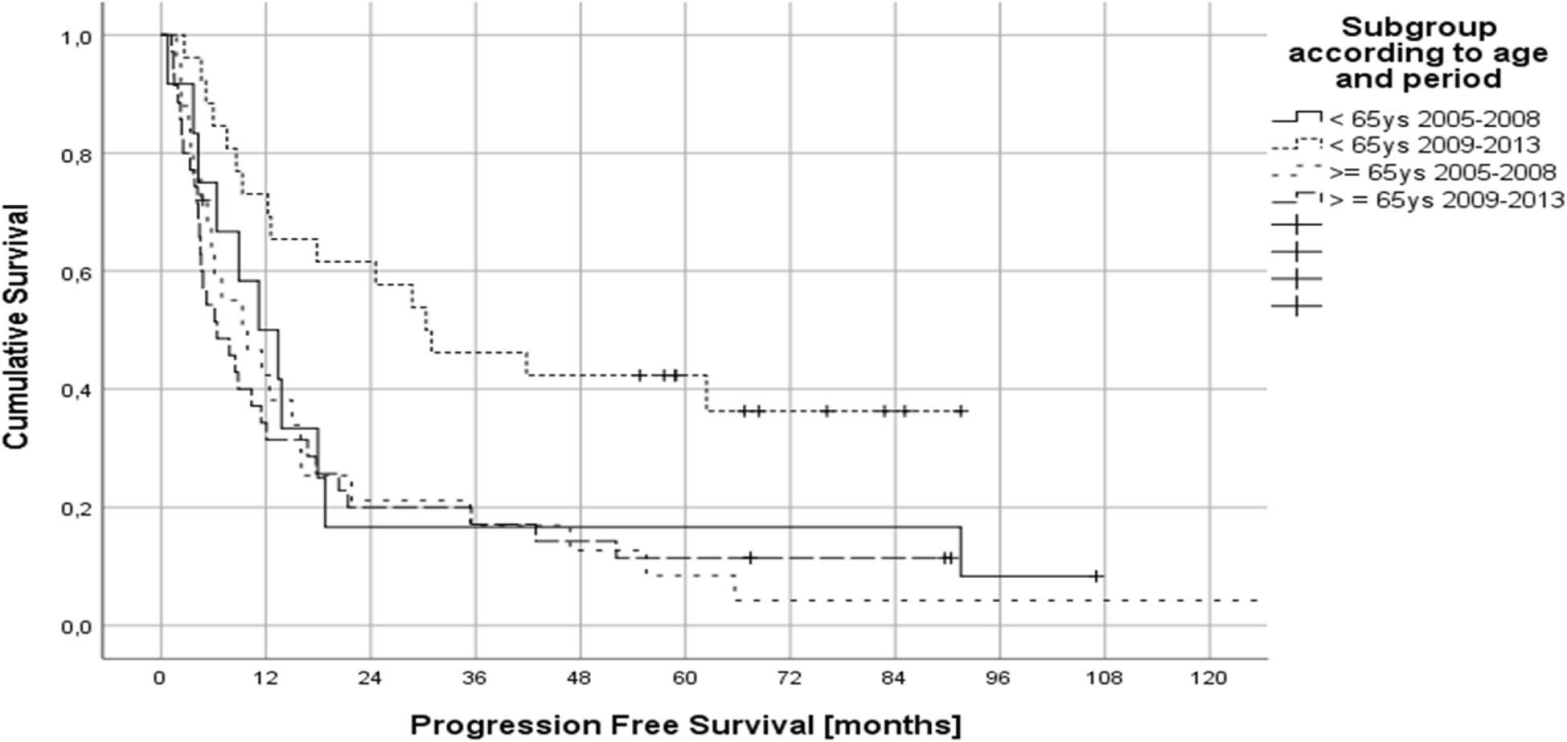
Progression free survival according to age and treatment period (group A: < 65 ys 2005–2008 received intrathecal liposomal Ara C; group B: < 65 ys 2009–2013 received intraventricular therapy via Ommaya reservoir [25/26 patients]; group C: ≥ 65 ys 2005–2008 received intrathecal liposomal Ara C; group D: ≥ 65 ys 2009–2013 received no intrathecal therapy [27/35 patients])

**Fig. 3:**
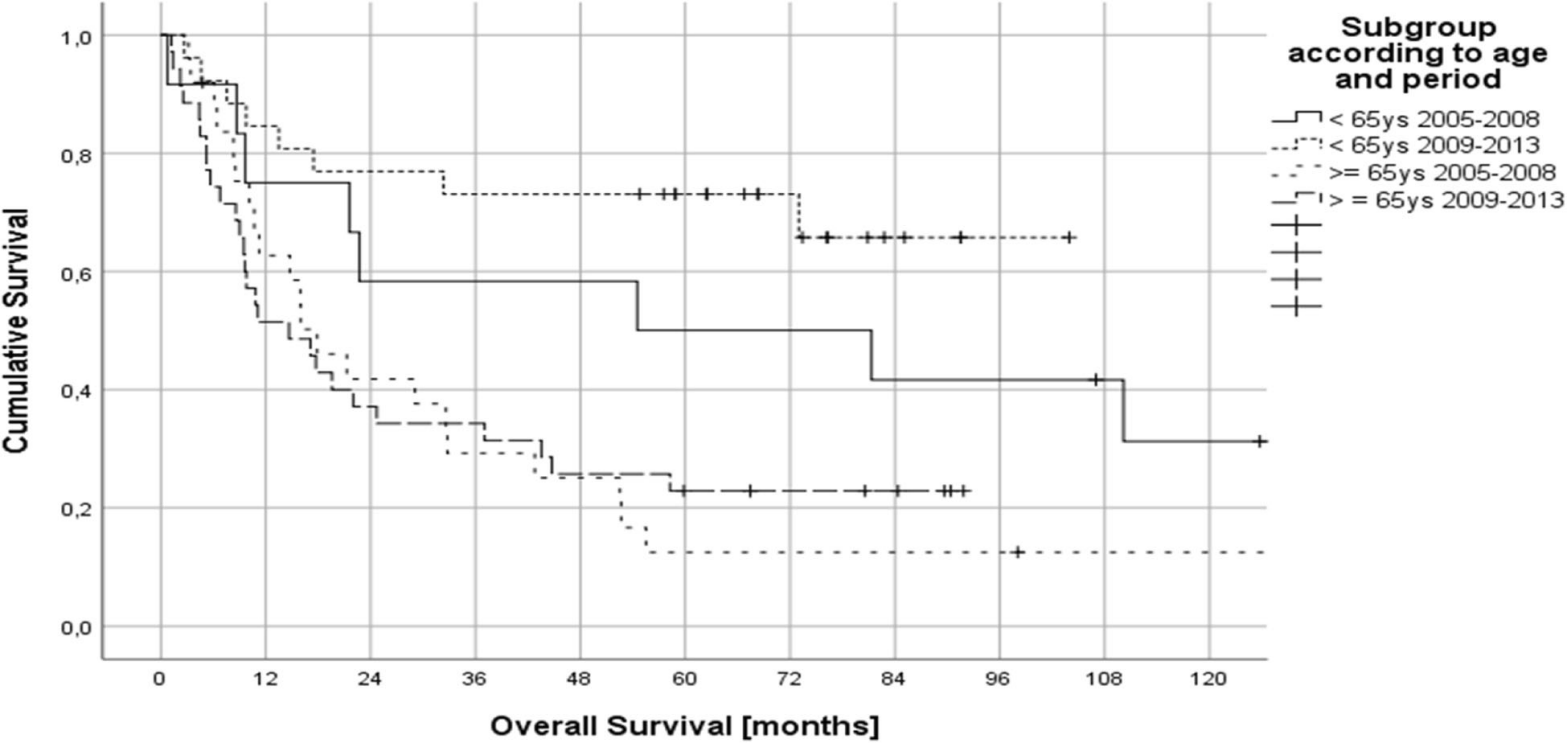
Overall survival according to age and treatment period (group A: < 65 ys 2005–2008 received liposomal Ara C i.th.; group B: < 65 ys 2009–2013 received i.c.v. therapy via Ommaya reservoir (25/26 patients); group C: ≥ 65 ys 2005–2008 received liposomal Ara C i.th; group D: ≥ 65 ys 2009–2013 received no i.th. therapy (27/35 patients)

Group A hat a median PFS of 11.2 months (95% CI 3.5–18.8), group B 30.3 months (95% CI 8.7–51.9), group C 9.8 months (95% CI 2.7–11.1) and group D 6.4 months (95% CI 2.1–10.7) (Fig. 2). Median OS was 54 months (95% CI 0–154) in group A, was not reached in group B, 17.9 months (95% CI 10.1–25.7) in group C and 14.6 months (95% CI 5.4–23.9) in group D (Fig. 3).

### Toxicity

Grade 3/4 toxicity is summarized in Additional file 1. Anemia, leucopenia, thrombopenia, infections and transaminases elevation were the most frequent grade 3/4 toxic side effects, occurring in 37, 54, 51, 41 and 44%, respectively. Of 37 patients treated with liposomal AraC intrathecally 7 (19%) developed grade 3 arachnoiditis. Of 33 patients treated with intraventricular chemotherapy via Ommaya reservoirs, reservoir infection occurred in three (9%) patients. In one patient the reservoir infection occurred immediately after implantation prior to application of intraventricular chemotherapy: the reservoir was removed, antibiotics were given, and after recovering a new reservoir was implanted and chemotherapy was continued as planned. In the two other patients the infection occurred 5 days after 1st cycle of intraventricular therapy and 6 days after 2nd cycle, respectively. In both the reservoir was removed, systemic antibiotics were given and chemotherapy was continued without intraventricular therapy. In one patient misplacement with extravasation of chemotherapy into the surrounding brain tissue occurred. In this patient, the reservoir was removed and the treatment was continued with systemic therapy only incl. HD-ASCT for consolidation. This patient experienced psychomotor slowing and disorientation, but fully recovered within 6 weeks. Six patients died on therapy, 4 of whom were ≥ 65 years. Reasons of death were myocardial infarction in one patient and infection in 5. Three of these patients suffered from pneumonia and for 2 patients the focus of infection could not be determined, but meningitis was ruled out.

### Prognostic factors

The following factors were tested: age, Karnofsky performance score (KPS), lactate dehydrogenase (LDH) in serum, cerebrospinal fluid (CSF) protein, involvement of deep brain structures, International Extranodal Lymphoma Study Group (IELSG) score and type of protocol (Additional files 2 and 3). On univariate analysis, younger age, higher KPS and lower IELSG score were associated with longer OS as were younger age and higher KPS with longer PFS. On multivariate analysis younger age and higher KPS were associated with longer PFS but only younger age was associated with longer OS: patients ≥65 years had a median PFS of 7.8 months (95% CI 4.4–11.2) and median OS of 16 months (95% CI 8.9–23.1), patients < 65 years had a median of PFS 17.9 months (95% CI 1.7–34.2) and median OS of 110 months (53.8–166.5). No significant influence on survival was found for LDH elevation in serum, protein elevation in the CSF and for involvement of deep brain structures.

When the influence of therapy on outcome was analyzed, a trend to better outcome for intraventricular therapy vs. intrathecal liposomal AraC was found in patients < 65 years: on univariate analysis HR was 0.53 (0.19–1.47) for OS and 0.46 (0.21–1.02) for PFS. No influence of rituximab on outcome was found.

Treatment at relapse is summarized in Additional file 4.

## Discussion

The current series represents a retrospective monocentric analysis. Yet the patient population analyzed in this series presumably reflects every day clinical practice of PCNSL treatment better than patient series treated within formal clinical trials, where patient selection is determined by stringent exclusion criteria. Except for severe organ dysfunction, representing contraindications to basic treatment elements, no such selection was carried out in the patient cohort presented here, such that 98/113 patients (87%) could be treated as intended. This is reflected by a relatively high median age of 67 years with 60% of patients ≥65 years in this series, which is in contrast to other studies using induction followed by consolidation with a median age of 51 to 60 years and 19 to 42% of patients > 60 years [10–13, 26–28]. Moreover, the KPS of patients in the present study was rather low with a median of 60 and 54% of patients with KPS < 70%, as compared to a median KPS of 70–90% and a percentage of 18 to 50 of patients with KPS < 70% in other studies [11, 12, 26, 27, 29]. This is to be considered when interpreting the current results.

As the Bonn protocol has proven to preserve neurocognitive function in long-term survivors [8, 30], since 2005 all patients with newly diagnosed PCNSL able to tolerate HDMTX at our center were treated with a protocol combining the elements of the Bonn protocol with the concept of induction-consolidation. In contrast to the original Bonn protocol, a 24 h infusion of HDMTX was replaced by a 4 h infusion, systemic drugs not able to cross the intact blood brain barrier (BBB) were omitted, cyclophosphamide was replaced by ifosfamide and intraventricular therapy was modified. Since several retrospective analyses suggested that rituximab could improve therapeutic efficacy in PCNSL [17, 18], rituximab was added to induction therapy in 2010.

When the results are compared to that achieved with the original Bonn protocol [6] similar response rates are found: CR/CRu of 60% vs. 61%, PR 9% vs. 10% and PD 23% vs. 19%. Moreover, similar 2-years OS was achieved: in patients < 61 years: 78% vs. 80% and in patients > 60 years: 41% versus 59%. When acute toxicity is compared, less grade 3–4 leukopenia and thrombocytopenia were found in the current analysis: 55% vs. 94 and 52% vs. 89%, respectively. The frequency of Ommaya reservoir infections which occurred in 19% of patients treated with the original Bonn protocol [6], was only 9% in the current series. This difference is not significant due to the small number of patients analyzed, but may reflect the fact, that intraventricular therapy is better tolerated in patients devoid of steroid treatment and in a good clinical condition. However, the frequency of grade 3 to 4 infections of 41% was higher than in the Bonn study with 26% [6], most likely as a consequence of a more frequently reduced general condition in the current series. The rate of toxic death was 6% in the current study as compared to 9% in the Bonn study. In our view, with the modifications made, the practicability of the Bonn protocol is improved, while the effectivity was preserved. When comparing the results achieved in this series with results of the original protocol it should be considered, that pre-therapeutic prognostic factors of the 65 patients included in the Bonn study were better than in the present study: the median age in the original Bonn study was 62 years with 54% of patients > 60 years, and median KPS was 70% with 25% of patients with KPS < 70%.

A particular emphasis of this analysis was put on the role of intrathecal therapy, with a focus on that of intraventricular treatment via an Ommaya reservoir. Comparing the results achieved with intrathecal therapies in patients < 65 years, our data suggest a higher efficacy of intraventricular therapy than of intrathecal liposomal AraC: On univariate analysis, an almost significant PFS difference was found benefitting intraventricular therapy with a HR of 0.46 (0.21–1.02), whereas the HR for OS was 0.53 (0.19–1.47). The number of patients presumably was too low to detect a difference of statistical significance. A potentially higher efficacy of intraventricular treatment with MTX, prednisolone and AraC in our view is not surprising. Liposomal AraC may well have advantageous pharmacokinetics in comparison to other formula, however, it has never been suggested, that MTX for systemic treatment in PCNSL may be replaced by AraC and this might be true for intrathecal therapy as well.

A high proportion of 91% of patients received salvage treatment at progression/relapse with systemic chemotherapy being the most frequent treatment, which is summarized in Additional file 4*.*

When the results of the present analysis are compared to other studies using the therapeutic concept of induction followed by consolidation, comparable results can be found at least. In those studies, CR-rates of 30–81% and PR-rates of 11–14% after completion of therapy were achieved despite younger age and better KPS as compared to this cohort [5, 10–13, 26–28]. Also, long term outcome in our series is in the higher range of the 2-years OS rates compared to other studies including elderly patients with 32–90% [5, 12, 26–28]. From our treatment benefitted particularly patients < 61 years whose 2-years PFS was 69%, 2-years OS 78% and 5-years OS 57%. This is in accordance to results by Morris et al. with 2-years PFS of 64% and DeAngelis et al. with 2-years PFS and –OS of approximately 75% each.

## Conclusions

This study has several limitations as it represents a retrospective monocentric analysis of a heterogeneously treated patient population with rather small subgroups.

Nevertheless, we considered the following possible therapeutic perspectives: HDMTX based induction followed by consolidation with intensive conventional chemotherapy plus intraventricular treatment can be applied to the vast majority of PCNSL patients with no upper age limit besides that set by possible comorbidity.

Intraventricular treatment via an Ommaya reservoir is associated with manageable complications of low frequency, if started with consolidation.

PCNSL treatment in elderly patients not qualifying for HD-ASCT is still extremely unsatisfying [29, 31–33]. Therefore, we currently apply intraventricular treatment as part of first-line therapy in every elderly patient beginning with consolidation as a valuable option of treatment intensification with no risk of additional systemic toxicity.

## Additional files

Additional file 1: CTC Grade 3–4 toxicity. (DOCX 15 kb)

Additional file 2: Influence of prognostic factors on Progression free survival. (DOCX 13 kb)

Additional file 3: Influence of prognostic factors on Overall survival. (DOCX 13 kb)

Additional file 4: Treatment at first relapse or in case of progressive disease during therapy (*n* = 68). (DOCX 13 kb)

## Abbreviations

BBB: Blood brain barrier
CR: Complete remission
CRu: Complete remission
CSF: Cerebrospinal fluid
HD AraC: High-dose cytarabine
HDMTX: High-dose methotrexate
HD-SCT: High dose chemotherapy with autologous stem cell transplantation
IELSG: International Extranodal Lymphoma Study Group score
KPS: Karnofsky performance score
LDH: Lactate dehydrogenase
OS: Overall survival
PCNSL: Primary central nervous system lymphoma
PD: Progressive disease
PFS: Progression free survival
PR: Partial remission
WBRT: Whole brain radiation therapy
