# Supplementary material for: HDMTX-based induction therapy followed by consolidation with conventional systemic chemotherapy and intraventricular therapy (modified Bonn protocol) in primary CNS lymphoma: a monocentric retrospective analysis
Source: Neurol Res Pract. 2019 Jun 20;1:17. doi: 10.1186/s42466-019-0024-2 (PMC7650117; doi:10.1186/s42466-019-0024-2)
Supplement: Supplementary file 1 — CTC Grade 3–4 toxicity. (DOCX 15 kb) [file 42466_2019_24_MOESM1_ESM.docx]

**Additional file 1:** CTC Grade 3-4 toxicity

|  | **Grade 3** | **Grade 4** |
| --- | --- | --- |
| Anemia | 31 (32%) | 6 (6%) |
| Leucopenia | 32 (33%) | 22 (22%) |
| Neutropenia | 14 (14%) | 35 (36%) |
| Lymphopenia | 23 (23%) | 72 (73%) |
| Thrombopenia | 33 (34%) | 18 (18%) |
| Infections  Ommaya-Inf. | 31 (32%)  3 (9%) | 9 (9%) |
| Urea or creatinine elevation | 2 (2%) | 1 (1%) |
| Aminotransferases elevation | 40 (41%) | 1. (3%) |
| Hyperglycemia  Hypokalimea | 2 (2%)  2 (2%) | 0  0 |
| Nausea  Vomiting  Diarrhea  Stomatitis | 3 (3%)  1 (1%)  1 (1%)  6 (6%) | 0  0  0  1 (1%) |
| Cardial ischemia  Tachyarrhythmia  Hypertension  Venous thrombosis | 1 (1%)  1 (1%)  1 (1%)  2 (2%) | 1 (1%)  0  0   1. (2%) |
| Dyspnea | 1 (1%) | 0 |
| Peripheral neuropathy  Headache  Impaired conciousness  Psychosis | 8 (8%)  1 (1%)  7 (7%)  1 (1%) |  |
| Skin | 2 (2%) |  |
