# Supplementary material for: HDMTX-based induction therapy followed by consolidation with conventional systemic chemotherapy and intraventricular therapy (modified Bonn protocol) in primary CNS lymphoma: a monocentric retrospective analysis
Source: Neurol Res Pract. 2019 Jun 20;1:17. doi: 10.1186/s42466-019-0024-2 (PMC7650117; doi:10.1186/s42466-019-0024-2)
Supplement: Supplementary file 2 — Influence of prognostic factors on Progression free survival. (DOCX 13 kb) [file 42466_2019_24_MOESM2_ESM.docx]

**Additional file 2:** Influence of prognostic factors on Progression free survival

|  | Univariate Analysis | | | Multivariate Analysis^1^ | | |
| --- | --- | --- | --- | --- | --- | --- |
|  | Hazard Ratio | 95% CI | p | Hazard Ratio | 95% CI | p |
| Age (< 65 vs. ≥ 65) | 2.02 | 1.27-3.22 | **0.003** | 1.94 | 1.21-3.12 | **0.006** |
| patients 2005-2008 vs. patients 2009-2013 | 0.71 | 0.46-1.11 | 0.134 | 0.81 | 0.52-1.28 | 0.37 |
| < 65 ys 2005-2008 vs. < 65 ys 2009-2013 | 0.46 | 0.21-1.02 | 0.055 | N.A.^2^ | N.A. | N.A. |
| ≥ 65 ys 2005-2008 vs. ≥ 65 ys 2009-2013 | 1.03 | 0.60-1.76 | 0.92 | N.A. | N.A. | N.A. |
| Karnofsky performance score | 1.78 | 1.14-1.16 | **0.011** | 1.63 | 1.04-2.55 | **0.035** |
| Lactate dehydrogenase in serum | 0.71 | 0.44-1.16 | 0.173 | 0.81 | 0.49-1.33 | 0.40 |
| Cerebrospinal fluid protein | 1.03 | 0.63-1.68 | 0.91 | 1.12 | 0.68-1.83 | 0.67 |
| Involvement Deep Brain Structures | 1.12 | 0.65-1.93 | 0.68 | 1.22 | 0.71-2.11 | 0.47 |
| IELSG-score^3^ | 1.46 | 0.99-2.16 | 0.059 | 1.19 | 0.77-1.85 | 0.44 |

^1^results refer to models with age plus one covariate, only Karnofsky Index was significant, ^2^no multivariate analysis due to stratification for age, ^3^IELSG categories derived from age and Karnofsky, thus high correlation with these variables
