# Supplementary material for: HDMTX-based induction therapy followed by consolidation with conventional systemic chemotherapy and intraventricular therapy (modified Bonn protocol) in primary CNS lymphoma: a monocentric retrospective analysis
Source: Neurol Res Pract. 2019 Jun 20;1:17. doi: 10.1186/s42466-019-0024-2 (PMC7650117; doi:10.1186/s42466-019-0024-2)
Supplement: Supplementary file 3 — Influence of prognostic factors on Overall survival. (DOCX 13 kb) [file 42466_2019_24_MOESM3_ESM.docx]

**Additional file 3:** Influence of prognostic factors on Overall survival

|  | Univariate Analysis | | | Multivariate Analysis^1^ | | |
| --- | --- | --- | --- | --- | --- | --- |
|  | Hazard Ratio | 95% CI | p | Hazard Ratio | 95% CI | P |
| Age (<65 vs. ≥ 65) | 2.87 | 1.64-5.02 | **<0.001** | 2.80 | 1.59-4.92 | **<0.001** |
| patients 2005-2008 vs. patients 2009-2013 | 0.72 | 0.44-1.18 | 0.19 | 0.81 | 0.49-1.34 | 0.41 |
| < 65 ys 2005-2008 vs. < 65 ys 2009-2013 | 0.53 | 0.19-1.47 | 0.22 | N.A.^2^ | N.A. | N.A. |
| ≥ 65 ys 2005-2008 vs. ≥ 65 ys 2009-2013 | 0.95 | 0.54-1.68 | 0.86 | N.A. | N.A. | N.A. |
| Karnofsky performance score | 1.73 | 1.05-2.84 | **0.03** | 1.56 | 0.94-2.57 | 0.083 |
| Lactate dehydrogenase in serum | 0.87 | 0.50-1.51 | 0.62 | 1.06 | 0.60-1.85 | 0.85 |
| Cerebrospinal fluid protein | 1.03 | 0.58-1.80 | 0.93 | 1.05 | 0.60-1.84 | 0.88 |
| Involvement Deep Brain Structures | 0.84 | 0.45-1.57 | 0.58 | 0.87 | 0.46-1.64 | 0.67 |
| IELSG-score^3^ | 1.72 | 1.09-2.71 | **0.02** | 1.39 | 0.85-2.27 | 0.185 |

^1^results refer to models with age plus one covariate, no additional covariate was significant, ^2^no multivariate analysis due to stratification for age, ^3^IELSG categories derived from age and Karnofsky, thus high correlation with these variables
