# Supplementary material for: HDMTX-based induction therapy followed by consolidation with conventional systemic chemotherapy and intraventricular therapy (modified Bonn protocol) in primary CNS lymphoma: a monocentric retrospective analysis
Source: Neurol Res Pract. 2019 Jun 20;1:17. doi: 10.1186/s42466-019-0024-2 (PMC7650117; doi:10.1186/s42466-019-0024-2)
Supplement: Supplementary file 4 — Treatment at first relapse or in case of progressive disease during therapy (n = 68). (DOCX 13 kb) [file 42466_2019_24_MOESM4_ESM.docx]

**Additional file 4:** Treatment at first relapse or in case of progressive disease during therapy (n=68)

| Treatment | Number of patients | < 65 years | ≥ 65 years |
| --- | --- | --- | --- |
| HD-SCT | n=25 | n=16 | n=9 |
| MTX-rechallenge | n=3 | n=1 | n=2 |
| Temozolomide | n=16 | n=1 | n=15 |
| Temsirolimus | n=1 | n=0 | n=1 |
| WBRT | n=8 | n=2 | n=6 |
| no treatment | n=6 | n=1 | n=5 |
| systemic relapse  (n=1 patient systemic and cerebral relapse)  5x R-CHOP  1x unknown | n=6 | n=1 | n=5 |
| ocular relapse  2x radiation of the ocular bulb  1x trophosphamide i.v. | n=3 | n=1 | n=2 |
|  | Total n=68 | Total n=23 | Total n=45 |
